# Mrs. Elizabeth Ann Renfrew Willoughby Cronyn

**Published:** 1912-02

**Authors:** 


Mrs. Elizabeth Ann Renfrew Willoughby Cronyn, widow of the late Dr. John Cronyn of Buffalo, died December 30, 1911, aged 89.

